# New procedures for the confirmation of brain death in Brazil: results from the *Central Estadual de Transplantes de Santa Catarina*

**DOI:** 10.5935/0103-507X.20210037

**Published:** 2021

**Authors:** Letícia Silva Wagner, Rafael Lisboa de Souza, Flávio Ricardo Liberali Magajewski

**Affiliations:** 1 Universidade do Sul de Santa Catarina, Campus Pedra Branca - Palhoça (SC), Brazil.; 2 Central Estadual de Transplantes de Santa Catarina, Secretaria de Estado da Saúde de Santa Catarina - Florianópolis (SC), Brazil.

**Keywords:** Brain death, Organ transplantation, Management indicators, Resolutions, Morte encefálica, Transplante de órgãos, Indicadores de gestão, Resoluções

## Abstract

**Objective:**

To analyze the impact of Resolution 2.173/2017 of the Federal Council of Medicine on results from the *Central Estadual de Transplantes de Santa Catarina*.

**Methods:**

This was a cross-sectional observational study of medical records of all patients (1,605) with suspected brain death notified to the *Central Estadual de Transplantes de Santa Catarina*; for this study, procedures to confirm this diagnosis were initiated between July 2016 and December 2017 and between January 2018 and June 2019. The median duration of the protocol in each period was considered for the comparison between the intervals. The collected data were transformed into rates (per million population). The mean rates for the periods before and after the implementation of the protocol were analyzed by Student’s t-test, and qualitative variables were analyzed by Pearson’s chi-squared test.

**Results:**

The mean duration of brain death confirmation procedures decreased more than 1 hour in the second period compared to the first period, with statistical significance (p = 0.001). The rates of harvested livers and transplanted pancreas, the number of notifications by hospital size and the rate of cardiac arrest in the macro-region of the Itajaí Valley were significantly different between the two periods.

**Conclusion:**

In the period after the new resolution on brain death, there was a reduction in the duration for diagnosis. However, other indicators did not change significantly, providing evidence for the multidimensional nature of the organ transplantation process in Santa Catarina and the need for further studies to better understand and optimize the process.

## Abstract (pt)

**Objetivo:**

Analisar o impacto da resolução 2.173/2017 do Conselho Federal de Medicina nos resultados da Central Estadual de Transplantes de Santa Catarina.

**Métodos:**

Estudo observacional do tipo transversal, com dados de prontuário de todos os pacientes (1.605) com suspeita de morte encefálica notificados à Central Estadual de Transplantes de Santa Catarina e que iniciaram procedimentos para confirmação desse diagnóstico entre julho de 2016 e dezembro de 2017 e entre janeiro de 2018 e junho de 2019. A mediana do tempo de duração do protocolo em cada período foi considerada para a comparação entre os intervalos. Os dados coletados foram transformados em taxas (por milhão de população). As taxas médias dos períodos antes e depois da implantação do protocolo foram analisadas pelo teste t de Student, e as variáveis qualitativas foram analisadas pelo teste do qui-quadrado de Pearson.

**Resultados:**

O tempo médio de duração dos procedimentos de confirmação de morte encefálica apresentou redução de mais de 1 hora no segundo período em relação ao primeiro, com significância estatística (p = 0,001). As taxas de fígados captados e de pâncreas transplantados, o número de notificações por porte hospitalar e a taxa de parada cardiorrespiratória na macrorregião do Vale do Itajaí também apresentaram diferenças com significância estatística na comparação entre os dois períodos.

**Conclusão:**

No período após a nova resolução sobre morte encefálica, houve redução do tempo de duração do diagnóstico. Contudo, outros indicadores não sofreram alteração significativa, evidenciando a natureza multidimensional do processo de transplante de órgãos em Santa Catarina e a necessidade de mais estudos para a melhor compreensão e otimização do processo.

## INTRODUCTION

In recent years, the notification rate of potential donors has gradually increased in Brazil;^(1,2)^ in 2016, there were 10,185 notifications, and 2,981 (29.3%) of these became effective donors.^(1)^ In the same year, there were 537 notifications in Santa Catarina, with 251 (46.7%) effective donors.^(3)^ In 2018, the total number of notifications in Brazil was 10,779, with 3,531 (32.7%) organ donations; in Santa Catarina, there were 581 notifications, with 287 (49.4%) effective organ donations.^(2.4)^

Brain death (BD) is defined as the state of non-perceptual coma, with the absence of supraspinal reactivity and persistent apnea in the presence of brain injury of known and irreversible cause.^(5)^ Currently, in Brazil, the criteria for the diagnosis of BD are defined by the Federal Council of Medicine (*Conselho Federal de Medicina* - CFM). The CFM published the first standard on the subject in 1991^(6)^ and revised the standards in 1997 through resolution 1,480.^(7)^ Resolution 1,480 was in effect for 20 years until resolution 2,173 was issued in December 2017,^(5)^ with some differences in relation to its predecessor.

Among the formalized changes, the following stand out: the minimum interval between clinical evaluations for patients older than 2 years decreased from 6 hours to 1 hour; only 1 positive apnea test was needed instead of 2 positive tests; and the test positivity parameter became a carbon dioxide (CO_2_) pressure greater than 55mmHg instead of greater than or equal to 55mmHg, as described in the 1997 resolution. In addition, some prerequisites were established that should be followed in a systematic and mandatory manner before performing each of the diagnostic steps: systolic blood pressure (SBP) ≥ 100mmHg or mean arterial pressure (MAP) ≥ 65mmHg, temperature > 35°C, and oxygen saturation > 94%. The new procedures also detailed morphological, organic, or acquired changes that preclude the unilateral evaluation of the reflexes and allow one to proceed to the next stages of diagnosis in case there are no reflexes on the side without changes. In addition, training criteria for medical examiners were defined, which replaced the requirement of a neurologist. Finally, the minimum time of in-hospital treatment from patient admission to the beginning of diagnostic procedures, which was not defined in the previous resolution, was established at 6 hours.^(5.7)^

In addition to ensuring that every critical patient has the right to a reliable diagnosis of death and reducing family doubts and suffering,^(8)^ transparency in the BD diagnosis has great effects on the organ donation and transplantation system. In this sense, the review of the BD diagnosis process was a recurring claim in Brazil.^(9)^ Pimenta et al. indicated that the cardiac arrest rate was 14% for patients whose interval between the 2 clinical tests for BD confirmation procedures was longer than 6 hours.^(8)^

Thus, evaluating the impact caused by CFM resolution 2,173/2017 is extremely important for implementing strategies that can speed up and make the BD diagnosis process increasingly safe; this reduces the suffering of families and increases the chance of transplantation to numerous patients in need of such procedures.

The objective of this study was to analyze the impact of CFM resolution 2,173/2017 on the management indicators of the *Central Estadual de Transplantes* (CET) of *Santa Catarina* between July 2016 and June 2019.

## METHODS

The protocol for this research project was submitted to the Ethics Committee on Research Involving Human Beings of the *Universidade do Sul de Santa Catarina* (UNISUL) under CAAE number 15650819.5.0000.5369 and was approved by opinion number 3.431.382.

This investigation was designed as a cross-sectional, analytical, observational study with a quantitative approach. Data sources were spreadsheets and individual medical records of all patients notified as suspected of BD in Santa Catarina, both under surveillance of the CET. The study population consisted of 1,605 patients for whom BD confirmation procedures began between July 2016 and June 2019 in 52 hospitals in Santa Catarina. Patients with contraindications and who had cardiac arrest (CA) before the completion of the protocol were excluded from the study; in addition, the medical records for 13 patients were not found through the Ethics Committee on Research Involving Human Beings due to an internal audit process under development during the collection period.

The variables evaluated were BD protocol opening notifications (number); sex (male/female); age (years); macro-region (North and Northeast Plateau/Greater Florianópolis/Vale do Itajaí/Rio Itajaí mouth/South/Midwest and Serra Catarinense/Grande Oeste); effective organ donations - those in which an organ was harvested (number); duration of the protocol (hours); opening of the protocol (time of day); end of the protocol (time of day); death by CA (yes/no); authorization from family members (yes/no); type of organ (kidney, liver, pancreas, heart and lung); hospital based on the Santa Catarina Hospital Policy, taking into account criteria such as number of beds, number of adult, pediatric and neonatal ICU beds, overall occupancy rate, number of clinics with hospitalization, available diagnostic tests, number of high complexities enabled and thematic network with authorized service^(10)^ (II/III/IV/V/unclassified); period (before, from July/2016 to December/2017; after, from January/2018 to June/2019) and month. The information collected was placed in a Microsoft Excel 2010 database, where the registration of a protocol was listed in rows and the details of each variable of interest for the study were listed in columns. The data were transformed into rates or coefficients; the numerator was the frequency of the protocols, and the denominator was the mean population of the state in the period considered. This ratio was multiplied by the constant 1 million (per million population - pmp). The mean rates for the period before and after deployment of the protocol described in CFM resolution 2,173/2017 were analyzed by Student’s t-test for independent samples. The qualitative variables were analyzed using Pearson’s chi-squared test. Values of p < 0.05 were considered significant.

## RESULTS

In total, 805 BD confirmation procedures were reported in the first period (July 2016 to December 2017), slightly higher than that reported in the second period (January 2018 to June 2019), i.e., 800 procedures; both periods together totaled 1,605 procedures studied.

The sociodemographic profile of patients undergoing the BD confirmation procedures in question are provided in table 1.

**Table 1:** Sociodemographic profile of patients undergoing brain death confirmation procedures

Variables	Period		p value
	2016 - 2017	2018 - 2019	
Protocols	805	800	
Age (years)	48.28 (± 19.42)	49.92 (± 18.13)	0.109
Sex			
Male	444 (55.2)	456 (57)	0.457
Female	361 (44.8)	344 (43)	
Hospital size			
II	8 (1)	7 (0.9)	
III	223 (27.7)	233 (29.1)	
IV	395 (49.1)	330 (41.3)	
V	144 (17.9)	173 (21.6)	
Not classified	35 (4.3)	57 (7.1)	0.007
Macro-region			
Northeast and North Plateau	192 (23.9)	193 (24.1)	
Greater Florianópolis	150 (18.6)	118 (14.8)	
Itajaí Valley	131 (16.3)	118 (14.8)	
Mouth of the Itajaí River	81 (10.1)	96 (12)	
South	97 (12)	107 (13.4)	
Midwest and Serra Catarinense	91 (11.3)	85 (10.6)	
Grande Oeste	63 (7.8)	83 (10.4)	0.163

Source: adapted from the *Central Estadual de Transplantes de Santa Catarina*.

The results are expressed as n, mean ± standard deviation or n (%).

Regarding the characterization of the two populations studied, the mean age and standard deviation in both periods were similar, as shown in table 1. The variation in sex prevalence between the first and second periods was not statistically significant (p = 0.457); however, there was a predominance (55.2% and 57%, respectively) of males in both cases.

Regarding the origin of the cases reported based on hospital size, in the second study period, there was a significant increase in the number of notifications from hospitals of a size not classified in the Santa Catarina Hospital Policy^(10)^. However, the hospitals classified as size II and IV reported fewer notifications in the second period than in the first period, while those classified as size III and V reported a higher number of notifications than in 2018. For the variable distribution of protocols based on the macro-regions of Santa Catarina, there was no significant change in the number of protocols open before and after the new CFM resolution.

The median duration of BD confirmation procedures (Table 2) decreased significantly in the second period (15.1 hours) compared to the first period (16.4 hours) (p = 0.001).

**Table 2:** Characterization of organ harvesting and donation processes, by study period

Variables	Period		Variation	p value
	2016 - 2017	2018 - 2019		
Time for BD confirmation (hours)	16,467 (1.58 - 224.7)	15,167 (1.01 - 227.7)	-7.83	0.001
Authorization from family members				
Yes	434 (66.7)	462 (69.5)	+2.8	
No	217 (33.3)	203 (30.5)	-2.8	0.275
Effective organ donation				
Yes	407 (93.1)	422 (90.9)	-2.2	
No	30 (6.9)	42 (9.1)	+2.2	0.226
CA				
Yes	77 (9.3)	59 (7.4)	-1.9	
No	728 (90.4)	741 (92.6)	+2.2	0.115
Organs harvested				
Liver	322 (79.3)	309 (72.9)	-6.4	0.030
Left kidney	391 (96.3)	404 (95.3)	-1.0	0.464
Right kidney	396 (97.5)	404 (95.3)	-2.2	0.082
Heart	27 (6.7)	37 (8.7)	+2.0	0.262
Pancreas	27 (6.7)	40 (9.4)	+2.7	0.141
Lung	23 (5.7)	25 (5.9)	+0.2	0.887
Organ transplantation				
Liver	262 (64.5)	247 (58.3)	-6.5	0.063
Left kidney	272 (67)	277 (65.3)	-1.7	0.612
Right kidney	268 (66)	273 (64.4)	-1.6	0.624
Heart	27 (6.7)	35 (8.3)	+1.6	0.379
Pancreas	15 (3.7)	31 (7.3)	+3.6	0.023
Lung	22 (5.4)	25 (5.9)	+0.5	0.766

Source: adapted from the *Central Estadual de Transplantes de Santa Catarina*.

BD - brain death; CA - cardiorespiratory arrest. The results expressed as n (shortest duration found - longest duration found) or n (%). Variation indicated as %.

The number of authorizations from family members increased from the first to the second period but was not statistically significant (p = 0.275); the same occurred for effective organ donation criterion (p = 0.226).

The loss rate due to CA decreased from 77 cases between 2016/2017 to 59 cases between 2018/2019 (-23.37%), but the difference was not statistically significant (p = 0.115).

Except for the liver, there was an increase the harvest of all other organs evaluated in this study in the second period compared to the first period, but the difference was not statistically significant (Table 2). Liver harvesting decreased considerably in the second period, from 322 to 309 harvested livers, a decrease that was statistically significant (p = 0.03).

The same occurred for transplanted organs, but in this case, the reduction in liver transplants was not significant (p = 0.063). However, the number of pancreas transplants increased significantly from 15 in the first period to 31 in the second period (p = 0.023).

Table 3 shows the productivity indicators (rates or coefficients) for the organ harvesting and transplantation system in relation to the average population of Santa Catarina for the two study periods. Regarding the rate of registered BD confirmation procedures, there was little difference between the periods studied (p = 0.764). The same occurred for the rate of effective organ donations (p = 0.828).

**Table 3:** Productivity rates per million population for the organ harvesting, donation, and transplantation system, by study period

Variables	Period		p value
	2016 - 2017	2018 - 2019	
Notification rate	76,986	74,904	0.583
Effective organ donation rate	38,923	39,512	0.828
CA rate per period	7,363	5,524	0.095
CA rate per macro-region			
North and Northeast Plateau	5,810	5,686	0.323
Greater Florianópolis	4,004	3,902	0.112
Itajaí Valley	1,888	1,846	0.029
Mouth of the Itajaí River	9,839	9,492	0.221
South	4,717	4,645	0.237
Midwest and Serra Catarinense	5,827	5,792	0.269
Grande Oeste	10,156	10,040	0.094
Organ donation rate per macro-region			
North and Northeast Plateau	48,417	45,962	0.715
Greater Florianópolis	40,614	41,257	0.924
Itajaí Valley	40,288	42,477	0.760
Mouth of the Itajaí River	51,165	56,953	0.571
South	33,695	35,835	0.753
Midwest and Serra Catarinense	34,966	28,962	0.378
Grande Oeste	18,620	23,428	0.419

Source: adapted from the *Central Estadual de Transplantes de Santa Catarina.*

CA - cardiorespiratory arrest.

The overall CA rate decreased in the second period, but the difference was not statistically significant (p = 0.095). The CA rates by macro-region of occurrence indicated that the Itajaí Valley was the only macro-region in which there was a significant reduction in the CA rate in the second period (p = 0.029). The other macro-regions also showed a reduction in the CA rate, but the difference was not statistically significant.

Finally, all values obtained for the organ donation rates per macro-region showed a p value > 0.05, indicating no statistically significant differences between the two periods studied.

## DISCUSSION

The aim of this study was to analyze the impact of CFM resolution 2,173/2017,^(5)^ in force since December 2017, on the management indicators of the CET of Santa Catarina between July 2016 and June 2019. The starting point for the study design was the following hypothesis: by reducing the period between the two clinical tests necessary for BD confirmation from 6 hours to 1 hour, the new procedures defined by the resolution would have a significant impact on losses due to CA, increasing the chance of effective organ donations and the supply of organs for transplantation.

Therefore, the time for BD confirmation diagnosis was one of the most important study indicators for the analysis of the impact of the new resolution and was used to confirm the initial hypothesis. The statistically significant reduction (>1 hour) in the median time for BD confirmation between the 2 periods indicated that the new procedures defined by resolution 2,173/2017 favored the performance of health teams in the diagnosis of BD among potential donors. Notably, during data collection, there was large variation in the duration of BD confirmation procedures, with the shortest lasting 1 hour and the longest lasting 227 hours. This distribution produced a logically improbable standard deviation when using the mean of the protocol times (standard deviation > mean value); therefore, the researchers chose to use the median times to reduce the effect of the aberrant values. One factor that can explain the extremely wide distribution for the duration of BD confirmation procedures is the availability of the necessary equipment to perform the mandatory graphic test for BD confirmation. There are different test options for this purpose: arteriography, transcranial Doppler, cerebral scintigraphy, and electroencephalogram. Each of these tests has its own particularities, and their availability in the state is heterogeneous, with the most distant regions along the coast, such as the macro-region of the Grande Oeste, having the fewest resources. According to Westphal et al.,^(11)^ graphical tests are responsible for considerably lengthening the protocol. The authors also note that Doppler is the most used modality in protocols with shorter durations. This aspect, which was not evaluated in this study, may have contributed to the production of this large difference between the duration of the procedures studied.

Another possible explanation for procedures with excessively long durations is the inclusion, in the statistics, of the production of hospitals that began to notify BD only after the new resolution was in effect. This possibility is supported by the difference in the distribution of hospitals based on size, as defined by the Santa Catarina Hospital Policy.^(10)^ In this sense, there was a remarkable decrease in open BD notifications in hospitals classified as size IV, and there was a significant increase in open BD notifications in hospitals classified as size V and hospitals whose size was not classified by the document. One cannot disregard the possibility that the increase in the number of notifications in hospitals whose size was not classified in the Santa Catarina Hospital Policy may be the result of expansion strategies of BD-notifying establishments and the incorporation of BD confirmation process improvements by inexperienced teams or by teams in establishments with less access to resources for confirmation using graphical tests.^(3,4,12,13)^

Considering that the widened standard deviation identified for the duration of the protocol in the second period could have been produced by notifying hospitals incorporating the new standard (n = 5), the analysis was repeated after excluding such hospitals to reduce possible bias produced by differentiated performance resulting from the learning curve for teams at these hospitals. The results showed no significant differences compared to the analysis already presented, indicating that the small production of these new notifying hospitals (only ten BD confirmation procedures after 2017) had no impact on the results of the first analysis. Thus, it was decided to present only the results for the two periods without any treatment in relation to the production of the notifying hospitals.

The following is an important aspect of the analysis of the results obtained: despite the reduction in the time for BD confirmation during the CFM resolution validity period, the total number of authorizations from family members did not change significantly. This finding can be explained by the small difference in the number of BD confirmation procedures completed in the two periods (651 family interviews conducted with family members in the first period and 656 interviews conducted in the second period). In this sense, it is necessary to emphasize that authorizations or refusals from family members for organ donation is a multicausal event and cannot be attributed only to the duration of the protocol.^(14-16)^ According to Pessoa et al.,^(14)^ the delay in diagnosis corresponded to only 10% of the reasons for refusal, and the most relevant aspect, observed in 21% of the cases studied, was the lack of understanding of BD by the family, followed by religious factors and the lack of technical competence of the team. In a way, the results obtained in this study from Santa Catarina corroborated the perspective that it is not possible to establish a firm correlation between the duration of BD confirmation procedures and the proportion of authorizations/refusals from family members for organ donation. The analysis by Lira et al.^(15)^ obtained similar results and highlighted the importance the team welcoming the family during the hospitalization process and the approach at the time of reporting death.

Additionally, it is necessary to consider that the processing of the death of the potential donor by the family is time-dependent, and a rapid diagnosis does not always result in greater number of authorizations from family members.^(16)^

One of the hypotheses of this study was that the possible reduction in the duration of BD confirmation procedures would have a relevant impact by reducing the loss of potential donors by CA. According to the study by Pimenta et al.^(8)^ performed in 2 hospitals in the city of Goiânia (GO) in 2011, under the old resolution, 14% of patients developed CA between the first and second clinical examinations, and for 16%, this occurred during the waiting period for the performance of complementary examinations. Considering that the minimum time interval between clinical examinations was 6 hours and decreased to 1 hour with CFM resolution 2,173/2017,^(5)^ which still allowed the complementary examination to be performed within this interval, a reduction in the number of losses by CA was expected. Despite the reduction observed (77 to 59 losses due to CA; 23.37% reduction), the decrease was not statistically significant. In addition, one should also consider that according to Westphal et al.,^(17)^ the care and maintenance of the potential donor in the initial period of BD, referred to by the author as a “sympathetic storm”, have relevant impacts on the hemodynamic stabilization of the potential donor and on the preservation of the quality of organs that can be donated; there is a loss of up to 20% of potential donors during this period, reinforcing the idea that the duration of BD diagnosis is not the only factor responsible for CA rates.^(18.19)^ In another study, the same author reported a lower prevalence of loss of potential donors due to CA between 12 and 30 hours since the beginning of the BD confirmation procedures, with an increase in this value before and after this interval.^(11)^

The CA rate has decreased in recent years following the greater structuring of the transplant system in Santa Catarina and in the country. In 2014, 13% of the BD notifications progressed to CA.^(20)^ In 2017, this proportion decreased to 8%,^(12)^ and according to the CET report, the proportion of losses due to CA in 2019 was 6.1%.^(13)^ The same is evidenced in the national context, with an increase in effective organ donations and a reduction in the number of CAs.^(21)^ These results suggest that the proportion of CA has tended to decrease over time and is not necessarily associated with a reduction in the duration of BD confirmation procedures.

Regarding the performance analysis of the system in relation to the number of organs harvested, there was a statistically significant reduction in liver harvesting, unlike what occurred in the country as a whole during the same period.^(21)^

In contrast, there was a significant increase in the number of transplanted pancreases in the second period studied. This finding is consistent with the results for the rest of the country, which also showed a growth trend since 2017.^(21)^ A possible explanation for these results may be the fact that the maximum ischemia time of this organ, which is 12 hours,^(22)^ makes it more sensitive to the duration of BD protocols. As a hypothesis, the reduction in the median time for BD confirmation allowed greater use of the organ.

When analyzing the performance of the macro-regions in relation to the indicators for the state transplantation system in the two periods studied, there was some discrepancy between the macro-regions, but the Itajaí Valley stood out both for a low CA rate and for a statistically significant difference between the 2 periods. In turn, Grande Oeste had a CA rate almost ten times higher than that of the Itajaí Valley, in addition to having a much lower organ donation rate. This finding highlights the heterogeneity found in the results for macro-regions and their relationship with the structures and resources available for the maintenance of potential donors, resulting in very different CA rates.^(18.19)^

In this sense, it is important to highlight the observed performance of the large hospitals in the Itajaí Valley Macro-region - *Hospital Santa Isabel*, in Blumenau, and the *Hospital Regional do Alto Vale*, in Rio do Sul - which showed increases in the number of notifications and organ donations in the study period associated with a reduction in the number of CAs. As an example, in 2017, despite Blumenau hospital being one of the hospitals with the most reports in the state, it did not record any loss due to CA, which may explain the statistically significant results obtained for the macro-region.^(3,4,12,13)^

The analysis of the results obtained in this study indicated that in the period of validity of CFM resolution 2,173/2017, there was a significant reduction in the median time for completion of the BD confirmation procedures, potentially contributing to improvements in the performance of Santa Catarina state in the last 2 years. Furthermore, clearer definitions of the prerequisites that enable the opening of the BD confirmation procedures, the criteria for training physicians responsible for diagnosis, and the enacting of CFM resolution 2,173/2017 may have contributed to the safety of the process, which was 1 of the motivations for updating the procedures studied.^(5)^ However, despite the reduction in the duration of BD confirmation procedures, the other indicators studied showed little difference between the 2 periods studied, and the results exposed the multifactorial nature of the process of diagnosis, organ donation, harvesting, and transplantation.

## CONCLUSION

The new procedures for the confirmation of brain death defined by Federal Council of Medicine resolution 2,173/2017 brought important additions to the diagnosis of brain death, with a relatively positive impact in Santa Catarina, i.e., a reduction in the time for diagnosis confirmation. However, indicators such as the cardiorespiratory arrest rate, the number of authorizations from family members, and the differences in organ donation among macro-regions did not change significantly with the new normative definitions. This lack of change shows the multidimensional nature of the organ donation, harvesting, and transplantation process in the state and the region and the need for further studies that can investigate this process, with approaches more appropriate to its objectives.
